# The R369 Myosin Residue within Loop 4 Is Critical for Actin Binding and Muscle Function in *Drosophila*

**DOI:** 10.3390/ijms23052533

**Published:** 2022-02-25

**Authors:** Adriana S. Trujillo, Karen H. Hsu, Meera C. Viswanathan, Anthony Cammarato, Sanford I. Bernstein

**Affiliations:** 1 Department of Biology, Molecular Biology Institute, Heart Institute, San Diego State University, San Diego, CA 92182, USA; atrujillo4@sdsu.edu (A.S.T.); khsu@sdsu.edu (K.H.H.); 2 Department of Medicine, Division of Cardiology, Johns Hopkins University, Baltimore, MD 21205, USA; madroxie1506@gmail.com (M.C.V.); acammar3@jhmi.edu (A.C.)

**Keywords:** myosin, myopathy, cardiomyopathy, *Drosophila melanogaster*, muscle

## Abstract

The myosin molecular motor interacts with actin filaments in an ATP-dependent manner to yield muscle contraction. Myosin heavy chain residue R369 is located within loop 4 at the actin-tropomyosin interface of myosin’s upper 50 kDa subdomain. To probe the importance of R369, we introduced a histidine mutation of that residue into *Drosophila* myosin and implemented an integrative approach to determine effects at the biochemical, cellular, and whole organism levels. Substituting the similarly charged but bulkier histidine residue reduces maximal actin binding in vitro without affecting myosin ATPase activity. R369H mutants exhibit impaired flight ability that is dominant in heterozygotes and progressive with age in homozygotes. Indirect flight muscle ultrastructure is normal in mutant homozygotes, suggesting that assembly defects or structural deterioration of myofibrils are not causative of reduced flight. Jump ability is also reduced in homozygotes. In contrast to these skeletal muscle defects, R369H mutants show normal heart ultrastructure and function, suggesting that this residue is differentially sensitive to perturbation in different myosin isoforms or muscle types. Overall, our findings indicate that R369 is an actin binding residue that is critical for myosin function in skeletal muscles, and suggest that more severe perturbations at this residue may cause human myopathies through a similar mechanism.

## 1. Introduction

Myosin II molecules incorporate into thick filaments in the sarcomeres of striated muscles and interact with disinhibited actin-containing thin filaments to produce force in an ATP-dependent fashion. Mutations that affect this interaction can cause striated muscle disease. For instance, the actin-binding site within the myosin motor domain of β-cardiac myosin (encoded by *MYH7*) is a hot-spot for mutations associated with dilated and hypertrophic cardiomyopathies (reviewed by Moore et al. [1]). *MYH7* mutations can also cause skeletal myopathies, since MYH7 is expressed in slow type 1 skeletal muscle fibers as well (reviewed by Oldfors [2]). Assessing interactions at the actomyosin interface may, therefore, yield insights into genetically-based skeletal myopathies and cardiomyopathies. 

**Figure 1:**
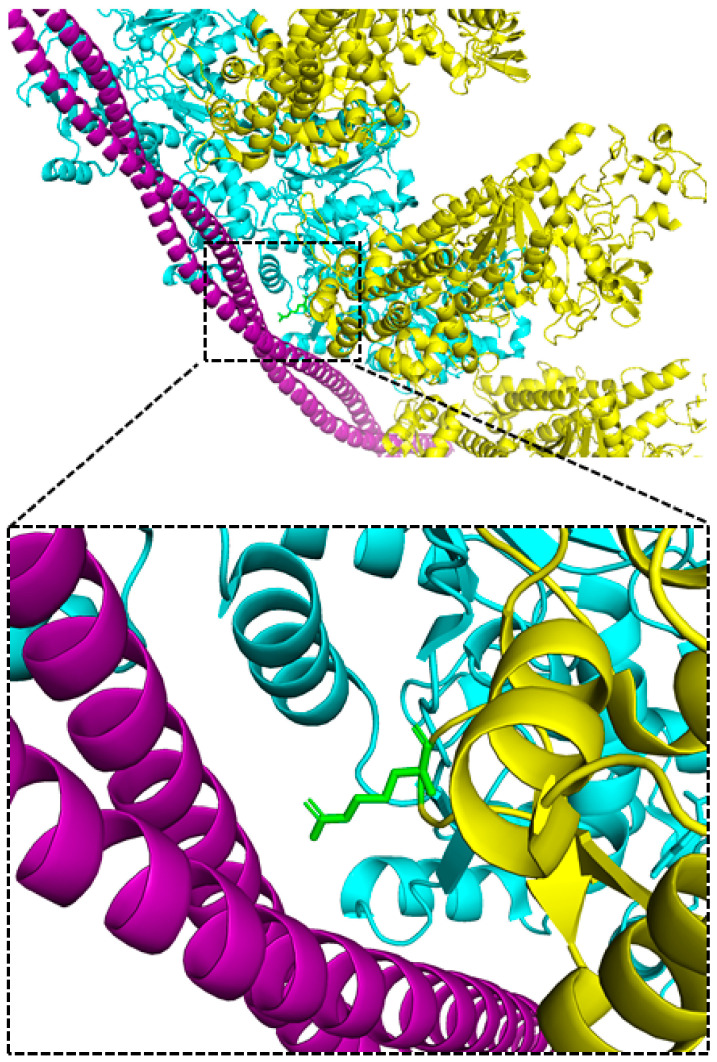
The R369 residue of myosin loop 4 resides at an interface of F-actin, myosin and tropomyosin (PDB: 6X5Z). The position of R369 (green) is shown within the structure of masseter β-myosin S1 (yellow) bound to *α*1-actin (cyan) and cardiac *αα*-tropomyosin (violet) solved by cryo-EM in conjunction with molecular docking of the following high-resolution structures [3]: the crystal structure of squid muscle myosin S1 in rigor (PDB: 3I5G), and cryo-EM structures of human cardiac F-actin (PDB: 6KN8), human cytoplasmic actomyosin (PDB: 5JLH), and rabbit skeletal muscle F-actin with mouse α-tropomyosin (PDB: 5JLF). The loop 4 densities for residues 354–380 were replaced with those of the crystal structure of β-cardiac myosin in the post-rigor state (PDB: 6FSA).

Myosin residue R369 (β-cardiac myosin amino acid numbering) resides on the tip of loop-4 of the upper 50 kDa subdomain and faces the outside of the molecule at an interface with actin and tropomyosin. The location of R369 is shown in a model of β-myosin S1 bound to F-actin and tropomyosin that was solved by cryo-electron microscopy (cryo-EM) and atomic-level molecular docking by Doran et al. [3] (Figure 1). Mutation of the R369 residue to histidine (R369H) occurs rarely, but represents the most common naturally-occurring mutation at that residue in multiple myosin isoforms of human skeletal muscles (Supplementary Table S1). Further, the β-myosin heavy chain arginine to glutamine mutation (R369Q) caused dilated cardiomyopathy in a pediatric patient [4], suggesting a critical conserved role of the residue in striated muscles.

Doran et al. proposed a model in which myosin R369 binds to tropomyosin to stabilize the transition from the C-state position (weak actomyosin binding) to the strong actomyosin binding M-state [3]. This model and other cryo-EM data suggest that β-myosin R369 is also directly involved in actin binding. Cryo-EM structures of actomyosin containing porcine cardiac β-myosin [5], bovine β-myosin [3], and *Dictyostelium* myosin-IE [6] show electrostatic interactions between loop 4 and actin. Further, molecular dynamics simulations incorporating the actomyosin cryo-EM complex in conjunction with X-ray structures for F-actin and chicken skeletal muscle myosin II predicted that myosin S1 binds actin at the R371 residue, which is homologous to β-cardiac myosin R369 [7]. Finally, in *Dictyostelium* myosin II, mutation of the entire loop containing this residue to glycine residues led to weakened actin binding [8].

We report here on the first (to our knowledge) transgenic model designed to examine the function of residue R369 in muscle tissue. Our model allowed us to test whether the R369H variant found in human skeletal muscle affects striated muscle structure and function in the fruit fly, *Drosophila melanogaster.* The R369H mutation (R368H in *Drosophila*) retains the positive charge of the wild-type residue, but introduces a bulky imidazole ring that may sterically hinder an electrostatic interaction with subdomain 3 of actin. Thus, we hypothesized that the R369H mutation would reduce actin binding and myosin function, leading to structural and functional defects in striated muscles. Our results demonstrate that the R369H mutation decreases myosin maximal binding to F-actin in vitro and reduces locomotion in adult *Drosophila*, indicating that R369 is critical to both actin interaction and muscle function. Defining the structural elements important for actomyosin interaction will yield further insight into the molecular mechanisms of myosin-induced myopathies and may allow for the development of targeted drug therapeutic treatments.

## 2. Results

### 2.1. The R369 Myosin Residue Modulates Actin Binding

We generated transgenic flies harboring the R369H mutation by *P* element transformation to determine the role of the R369 residue in regulating actomyosin interactions. Multiple fly lines were produced to ensure reproducibility of in vivo functional analyses. We crossed the transgenes into the indirect flight muscle (IFM) and jump muscle myosin-null *Mhc*^10^ line [9] and selected fly lines with wild-type myosin to actin ratios, as determined by polyacrylamide gel electrophoresis analysis of the upper thoraces of young mutant flies (mean ± S.E.M: 95.3 ± 2.4, 105.4 ± 3.4, and 104.6 ± 2.1 for *R369H* lines *L1*, *L2*, and *L3*, respectively, relative to wild-type *yw* flies). RT-PCR (reverse transcription polymerase chain reaction) of RNA isolated from IFM lysates of these lines confirmed the expression of the mutation and the absence of endogenous myosin heavy chain expression.

**Figure 2:**
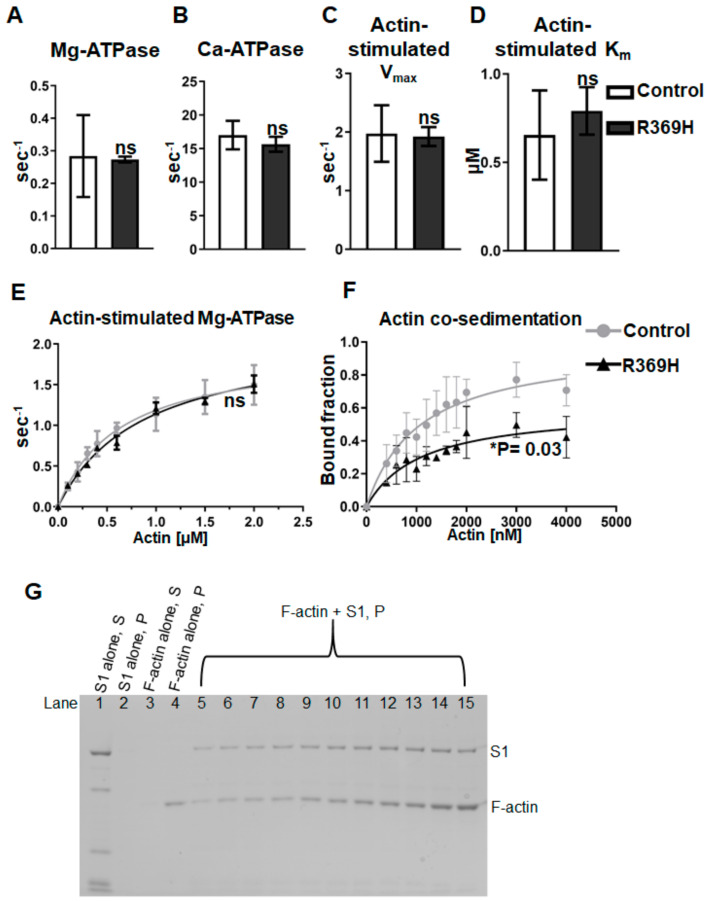
R369H myosin reduces maximal actin binding without affecting ATPase activity. (**A**–**E**) Full-length myosin isolated from indirect flight muscle (IFM) of *R369H* mutants (N = 3) and *PwMhc2* wild-type controls (N = 4) was assessed for the following ATPase parameters: (**A**) Mg^2+^ basal ATPase activity, (**B**) Ca^2+^ ATPase activity, (**C**) *V_max_* of actin-stimulated Mg^2+^ ATPase, and (**D**) actin affinity relative to ATPase (*K_m_*). (**E**) To determine actin-stimulated activity, Mg^2+^ basal ATPase activities were subtracted from measured ATPase values without actin and over increasing concentrations of F-actin. Data were fit with the Michaelis–Menten equation to determine *V_max_* and *K_m_*_._ Basal and actin-activated ATPase parameters are unchanged in R369H myosin compared to control myosin. (**F**) Actin co-sedimentation experiments revealed that the R369H mutation reduces the maximal binding (*B_max_*) of F-actin (N = 3). Bound myosin S1 fractions in actomyosin samples were determined via densitometry and plotted versus F-actin concentration. Bound fractions were calculated as the density of S1 in the insoluble pellet fraction relative to total protein content minus the fraction of pelleted S1 in a control containing S1-alone. The actin-binding dissociation constant for S1 (*K_d_*) was defined as the concentration of F-actin required to reach half *B_max_*. For all assays, data are reported as mean ± SD (see text for specific values). Statistical significance was determined using Student’s *t*-tests (ns = not significant; * *p* ≤ 0.05). (**G**) A representative SDS-polyacrylamide gel containing insoluble pellet (P) fractions of R369H S1 and F-actin-containing samples over increasing concentrations of F-actin (Lanes 5–15: 0.4, 0.6, 0.8, 1, 1.2, 1.4, 1.6, 1.8, 2, 3, and 4 μM). Supernatant (S) and pellet (P) fractions from S1-alone or F-actin-alone controls are shown (Lanes 1–4).

Steady state ATPase parameters of myosins prepared from dissected IFMs were assessed to determine if the R369H mutation affects the enzymatic activity of myosin (Figure 2). Ca-ATPase as well as basal Mg-ATPase activities did not significantly differ between R369H and transgenic wild-type (PwMhc2) myosin (Figure 2A,B). Furthermore, the *V_max_* (maximum ATPase rate) of actin-stimulation, as well as the affinity constant (*K_m_*) of actin required to reach 50% *V_max_* did not differ for R369H relative to control myosin (Figure 2C–E).

To determine whether the R369 residue modulates actin binding, we measured the binding affinity of R369H S1 for F-actin using actin cosedimentation. To obtain adequate levels of protein without the need for dissections, we generated a transgenic fly line expressing His-tagged myosin in IFMs and purified the His-tagged protein from fly homogenates using Ni column chromatography [10]. We then purified the subfragment-1 (S1) motor domain using limited proteolysis and size exclusion chromatography. F-actin was incubated with a fixed concentration of control or R369H S1 under rigor conditions (in the absence of nucleotide). Following high speed centrifugation, S1 remained soluble in S1-only (actin-free) controls, while F-actin was insoluble in F-actin-only (S1-free) controls (Figure 2G). SDS-polyacrylamide electrophoresis gels of centrifuged samples were scanned and densities of S1 bound fractions were fitted to a hyperbolic function to determine the maximal binding (*B_max_*) and dissociation constant (*K_d_*) for F-actin (Figure 2F). R369H S1 exhibited a significantly lower *B_max_* value compared to control S1 (0.597 ± 0.085 vs. 1.024 ± 0.211, *p* = 0.03), indicating reduced maximal binding to F-actin in vitro. Values for *K_d_* did not significantly differ for mutants compared to controls (1100 ± 510 nM vs. 1260 ± 963 nM, *p* = 0.81). 

### 2.2. The R369H Myosin Mutation Reduces Locomotion without Affecting Skeletal Muscle Ultrastructure in Drosophila

**Figure 3:**
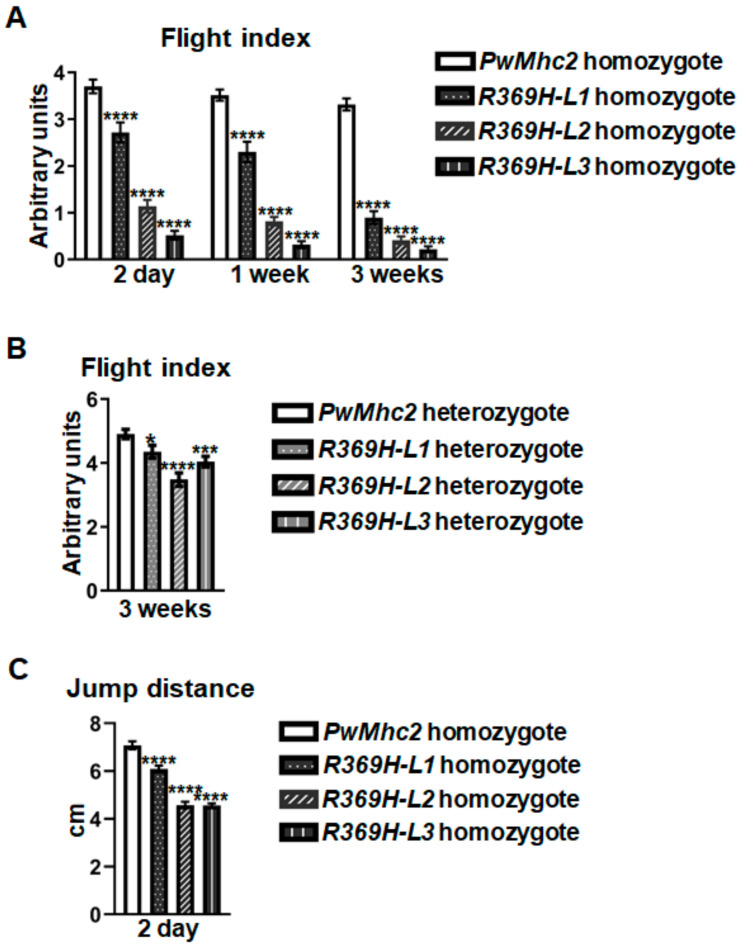
The R369H myosin mutation reduces locomotion in *Drosophila.* *R369H* mutant lines (*L1*, *L2*, and *L3*) were crossed into a *Mhc^10^* background that is null for endogenous myosin in IFMs and jump muscles. (**A**,**B**) Flight indices were calculated as 6*U/T +4*H/T +2*D/T +0*N/T, where each fly was scored for flight upward (U), horizontally (H), downward (D) or the inability to fly (N), and T represents the total number of flies tested. N ≥ 100 flies for each line/age. (**A**) Homozygous mutants exhibit declines in flight ability that are progressive with age. The statistical significance of differences between groups arising from effects of age and genotype was determined using a two-way ANOVA (Age: *p* < 0.0001, Genotype: *p* < 0.0001, Interaction: *p* < 0.0001). Multiple comparisons between mutants and *PwMhc2* controls are shown (**** *p* < 0.0001). (**B**) Heterozygous mutants exhibit reduced flight ability at 3 weeks of age. A one-way ANOVA determined statistical significance between genotypes (* *p* ≤ 0.05, *** *p* < 0.001, **** *p* < 0.0001). (**C**) Jumping distances are reduced in 2-day-old homozygous mutants. The average values of the top 3 of 10 jump distances are reported (N = 40 per line). A one-way ANOVA determined statistical significance of differences between genotypes (**** *p* < 0.0001). Values represent mean ±S.E.M. Full genotypes are defined as: *R369H* homozygotes (*Mhc^1^*^0^/*Mhc^1^*^0^; *P[R369H]*/*P[R369H]*), *PwMhc2* homozygotes (*P[PwMhc2]*/*P[PwMhc2]; Mhc^1^*^0^/*Mhc^1^*^0^), *R369H* heterozygotes (*Mhc^10^/*+; *P[R369H]/-*), and *PwMhc2* heterozygotes (*P[PwMhc2]/-*; *Mhc^10^/*+), where “-” indicates the absence of a transgene on the homologous chromosome.

To assess whether the R369H mutation affects fly locomotion, we measured flight and jump abilities of mutant and *PwMhc2* (*P* element wild-type controls) lines. Homozygous mutant lines (*L1*–*L3*) displayed reduced flight ability compared to controls at ages 2 days [arbitrary units (A.U.) ± S.E.M.]: 2.72 ± 0.21, 1.14 ± 0.14, and 0.52 ± 0.09 vs. 3.70 ± 0.15 A.U.], 1 week (2.30 ± 0.22, 0.82 ± 0.09, and 0.32 ± 0.07 vs. 3.52 ± 0.12 A.U.), and 3 weeks (0.90 ± 0.14, 0.42 ± 0.08, and 0.22 ± 0.06 vs. 3.32 ± 0.13 A.U.) (Figure 3A). Two-way ANOVAs determined that age (*p* < 0.0001) and genotype (*p* < 0.0001) as two independent factors, as well as their interaction, contribute to statistically significant differences between each group. Thus, the R369H mutation causes defects in flight ability that are progressive with age. Heterozygous lines (*L1–L3*) display significant reductions in flight ability compared to controls at 3 weeks of age (4.35 ± 0.20, 3.48 ± 0.21, and 4.04 ± 0.17 vs. 4.91 ± 0.15 A.U.; *p* = 0.0243, *p* < 0.0001, and *p* = 0.0004) (Figure 3B), suggesting that the detrimental effects of the mutation on locomotion are dominant. Mean jump distances were also reduced in all three homozygous mutant lines (*L1*, *L2*, and *L3*) compared to controls at 2 days of age (6.09 ± 0.14, 4.58 ± 0.14, and 4.56 ± 0.09 cm vs. 7.08 ± 0.16 cm, *p* < 0.0001) (Figure 3C).

**Figure 4:**
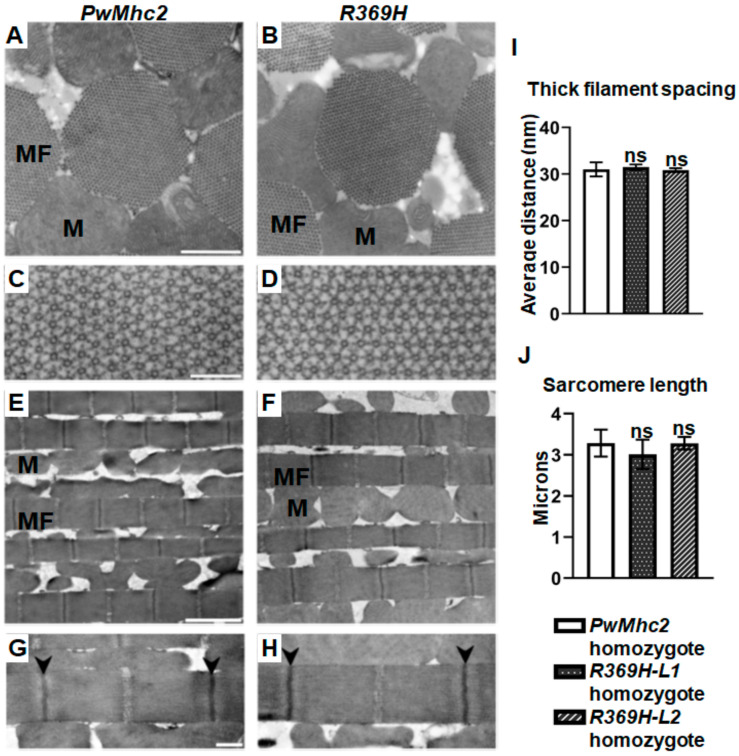
R369H myosin does not affect the stability of IFM myofibrils. Transmission electron micrographs of thin-sectioned IFMs in longitudinal orientation were obtained from 3-week-old homozygous *PwMhc2* control or *R369H* mutant flies in a *Mhc^1^*^0^ background that is null for endogenous myosin in IFMs and jump muscles. (**A**,**B**) Low-magnification micrographs of transverse sections show that the morphology of IFM myofibrils is normal in *R369H* mutant flies compared to controls. Scale bar, 0.5 µm. (**C**,**D**) High-magnification micrographs of transverse sections show a normal hexagonal pattern of thick and thin filaments in mutants and controls. Scale bar, 0.1 µm. (**E**,**F**) Low magnification images of myofibrils in longitudinal orientation show normal myofibrillar organization in *R369H* mutant IFMs. Scale bar, 2 μm. (**G**,**H**) High magnification images of longitudinal sections show normal sarcomeres bordered by Z-disks (arrowheads) in mutants. Scale bar, 0.5 μm. (**I**) Mean inter–thick filament spacing values were determined from images of transverse sections using a custom Python script. ≥700 thick filament centers per biological replicate (N = 3). (**J**) Mean sarcomere lengths were calculated from images of longitudinal sections using Image-J software. Fifty sarcomeres, N = 3 for each line. Data are reported as mean ± SD. Statistical significance was determined using Student’s *t*-tests, where ns = non-significant difference compared to controls. MF: myofibril, M: mitochondrion. Full genotypes are defined as follows: *R369H* homozygotes (*Mhc*^10^/*Mhc^1^*^0^; *P[R369H]*/*P[R369H]*); *PwMhc2* homozygotes (*P[PwMhc2]/P[PwMhc2]; Mhc^1^*^0^/*Mhc^1^*^0^).

We performed transmission electron microscopy on thin-sectioned IFMs from 3-week-old *R369H* homozygous mutants and controls to determine the ultrastructural effects of the mutation. Myofibrils of mutants and controls display a normal, rounded morphology in transverse sections (Figure 4A,B), and a double hexagonal pattern of thick and thin filaments (Figure 4C,D) that is characteristic of *Drosophila* IFMs [11,12,13]. In longitudinal sections, myofibrils are well-organized in a parallel fashion (Figure 4E,F) and contain normal sarcomeres with intact myofilaments (Figure 4G,H). Average inter-thick filament distances (Figure 4I) and sarcomere lengths (Figure 4J) did not differ between mutants and controls. Overall, IFM ultrastructure was not disrupted in *R369H* mutants, suggesting that flight impairment in these lines was not caused by defects in myofibrillar assembly or maintenance.

### 2.3. The R369H Myosin Mutation Does Not Affect Contractility, Dynamics or Ultrastructure of the Heart in Drosophila

**Figure 5:**
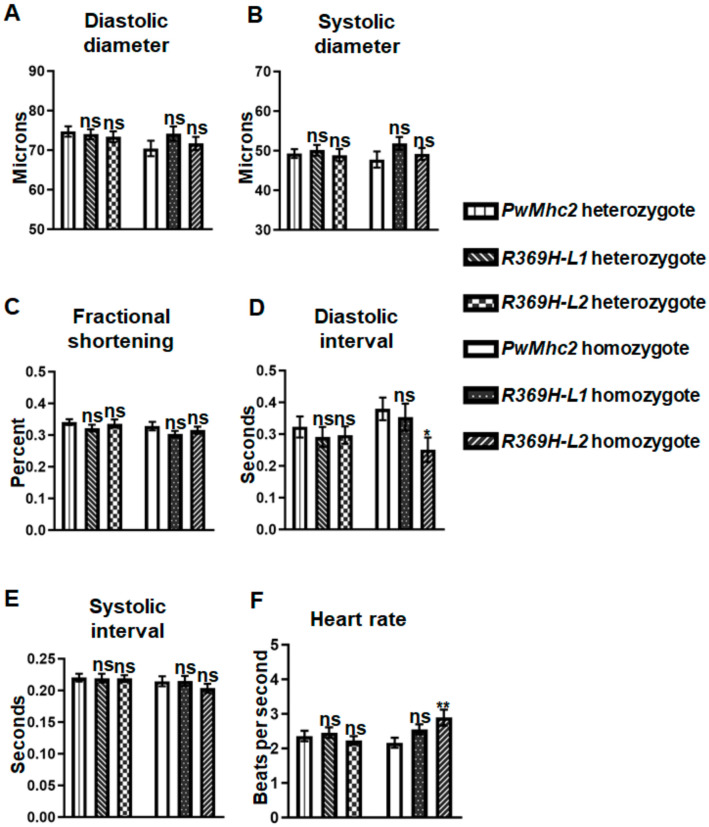
The R369H mutation does not affect cardiac physiological parameters in *Drosophila*. Homozygous or heterozygous mutant *R369H* lines (*L1* or *L2*) and *PwMhc2* controls were crossed into a myosin-null *Mhc*^1*/*^*Mhc*^1^ *or Mhc*^1*/*^*+* background, respectively. (**A**,**B**) Cardiac diameters and (**C**) fractional shortening values do not differ in 4-day-old homozygous and heterozygous lines compared to controls. *R369H-L2* homozygous flies display (**D**) reduced diastolic intervals and (**F**) elevated heart rates, while *R369H-L1* homozygous flies display no differences in cardiac dynamics (**D**–**F**) compared to controls. N ≥ 30 flies for each genotype. Values represent mean ± S.E.M. One-way ANOVAs determined statistical significance compared to controls (* *p* ≤ 0.05, ** *p* < 0.01, and ns = non-significant). Full genotypes are defined as follows: *R369H* homozygotes (*Mhc^1^*/*Mhc^1^*; *P[R369H]*/*P[R369H]*); *PwMhc2* homozygotes (*P[PwMhc2]/P[PwMhc2]; Mhc^1^*/*Mhc^1^*); *R369H* heterozygotes (*Mhc^1^/*+; *P[R369H]/-*); and *PwMhc2/+* heterozygotes (*P[PwMhc2]/-*; *Mhc^1^/*+), where “-” indicates the absence of a transgene on the homologous chromosome.

To determine whether the R369H mutation affects cardiac contractility or dynamics, we crossed mutant *R369H* and control *PwMhc2* lines into the *Mhc^1^* myosin null genetic background [14]. Since homozygous *PwMhc2* controls in the *Mhc^1^* background have an eclosion rate that is reduced by −50% and a median lifespan of 9 days [11], only young 4-day-old homozygous flies were examined. Heterozygous *R369H/+* and homozygous *R369H*/*R369H* mutant flies showed no differences in diastolic (DD) and systolic (SD) diameters, as well as fractional shortening (FS), compared to controls at 4 days of age (Figure 5A–C). *R369H-L2* homozygotes showed a higher heart rate compared to controls (Figure 5F) (2.89 ± 0.23 vs 2.17 ± 0.14 beats/s, *p* = 0.0018) caused by a reduction in diastolic intervals (Figure 5D) (0.25 ± 0.04 vs. 0.38 ± 0.04 s, *p* = 0.0117). However, line *R369H-L1* showed no differences in cardiac dynamics compared to controls (Figure 5D–F). *R369H/+* heterozygotes showed no differences in diastolic diameters (67.48 ± 1.51, *p* = 0.89 and 65.27 ± 1.46, *p* = 0.33 vs. 67.21 ± 1.26 μm), systolic diameters (43.73 ± 1.26, *p* = 0.47 and 41.88 ± 0.99, *p* = 0.07 vs. 44.93 ± 1.27 μm), or fractional shortening (0.35 ± 0.01, *p* = 0.29 and 0.36 ± 0.01, *p* = 0.13 vs. 0.33 ± 0.01) compared to controls at 3 weeks of age, suggesting that expression of the R369H mutation does not cause dominant reductions in cardiac contractility.

**Figure 6:**
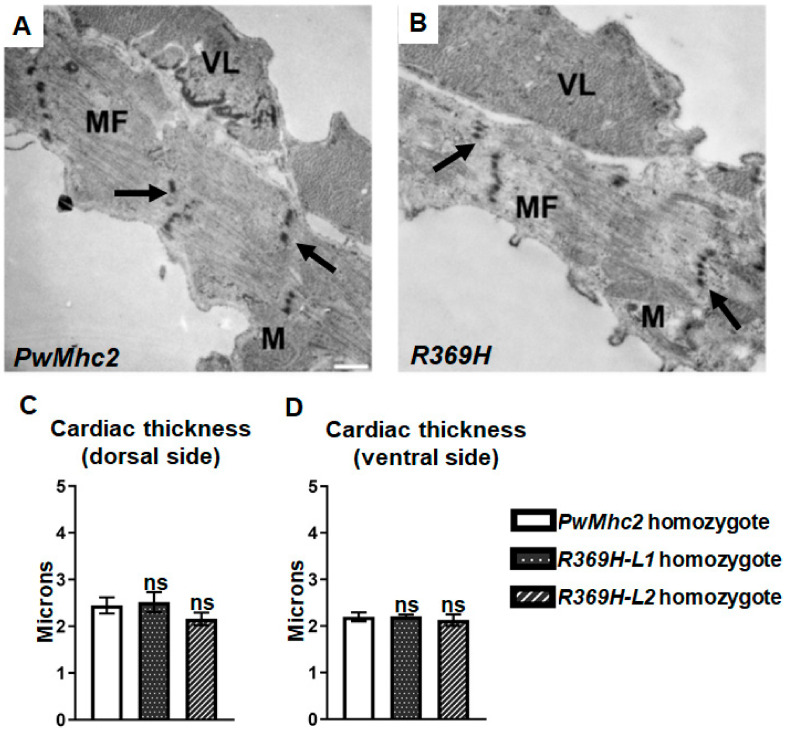
The R369H mutation does not affect assembly and stability of cardiac myofibrils in *Drosophila.* Homozygous mutant *R369H* lines (*L1* or *L2*) and *PwMhc2* controls were crossed into a myosin-null *Mhc^1/^Mhc^1^* background. (**A**,**B**) Transmission electron micrographs of transverse sections of the heart between the 3rd and 4th sets of ostia show no differences in the organization or stability of myofibrils in 4-day-old *R369H* homozygous lines compared to controls. The arrows indicate that Z-disks are discontinuous, as standardly observed in *Drosophila* cardiac myofibrils [11]. MF: myofibril. M: mitochondrion. VL: ventral-longitudinal skeletal muscle fibers. Scale bar, 0.5 µm. (**C**,**D**) Cardiac thickness values of dorsal-side (**C**) and ventral-side (**D**) areas of the heart do not differ in *R369H* lines compared to controls (N = 3 for each line). Values represent mean ± SD. Statistical significance was assessed using Student’s *t* tests, where ns = non-significant. Full genotypes are defined as follows: *R369H* homozygotes (*Mhc^1^*/*Mhc^1^*; *P[R369H]*/*P[R369H]*); *PwMhc2* homozygotes (*P[PwMhc2]/P[PwMhc2]; Mhc^1^*/*Mhc^1^*).

Finally, we determined whether the R369H mutation affects heart ultrastructure using transmission electron microscopy. For both the mutant and control homozygotes, myofibrils are oriented in a circumferential array around the heart tube and contain discontinuous Z-disks (Figure 6A,B, arrows), as previously reported in this tissue [11,15,16,17,18,19]. Mean cardiac thickness values on the dorsal-side and ventral-side do not differ in 4-day-old homozygous mutants compared to controls (Figure 6C,D). Overall, myofibrillar assembly and maintenance in cardiomyocytes appear unaffected by the mutation.

## 3. Discussion

Here we introduced a conservative R369H mutation into muscle myosin in the *Drosophila* model system to probe the role of this amino acid residue in regulating myosin function at the biochemical, ultrastructural and physiological levels. This residue is located within loop 4 of myosin, within its upper 50 kDa subdomain [8,20]. Previous cryo-EM work revealed that loop 4 is one of multiple surface loops at the actomyosin interface [7,21,22]. The R369H mutation introduces a bulky imidazole ring which may sterically hinder interaction with actin.

We found that R369H myosin isolated from IFMs displayed reduced maximal binding to F-actin in vitro (Figure 2F,G), confirming that the R369 residue modulates actin binding. During the crossbridge cycle, weak binding of myosin to actin triggers conformational changes that promote the release of the products of ATP hydrolysis [inorganic phosphate (P_i_), followed by ADP] [23]. When P_i_ is released, further intermolecular interactions at the actin interface stabilize a state in which myosin is strongly bound to actin [23,24,25]. These events are coupled with two lever arm movements (power stroke steps) that induce force generation.

We observed no change in ATPase parameters of *Drosophila* R369H myosin relative to control myosin (Figure 2A–E). The *K_m_* of actin affinity relative to ATPase is unchanged, consistent with our co-sedimentation data that the actin binding affinity (*K_d_*) does not differ relative to controls. Thus, it appears that the transition from weak to strong actin binding is not disrupted by the mutation. Since the *B_max_* of actin co-sedimentation is reduced in the mutant, it is possible that the mutation disrupts interactions important for stabilizing the strong actin binding state(s). In support of this hypothesis, a cryo-EM model of actomyosin incorporating myosin crystal structures showed that loop 4 directly interacts with actin in the strongly-bound rigor state, but not when myosin is weakly bound to actin [24]. Future approaches can employ transient kinetics analyses to determine the exact chemomechanical steps affected by the R369H mutation.

Biochemical studies in other systems add support to the hypothesis that loop 4 plays a critical role in binding actin. Proteolytic cleavage of loop 4 reduced actin affinity >100-fold in bovine β-cardiac myosin [26]. Further, fluorescence and kinetic measurements revealed that the mutation of loop 4 residues to glycine reduced actin binding in *Dictyostelium* myosin II [8]. Additionally, the mutation of a loop 4 G362 residue to alanine reduced actin affinity in chicken gizzard smooth muscle heavy meromyosin [20].

An interaction between loop 4 and actin is supported by both cryo-EM and computational work, where evidence suggests that residue R369 is critical to this interface. Cryo-EM structures of skeletal muscle myosin II [24] and myosin V [27] bound to F-actin show direct electrostatic interactions between loop 4 and actin, though loop 4 of myosin V localizes closer towards the cardiomyopathy loop where it forms an intramolecular hydrogen bond. A molecular dynamics simulation [7] of a cryo-EM structure of actomyosin [21] incorporating the myosin II [28] and myosin V [29] crystal structures, revealed that the residue homologous to β-cardiac myosin R369 (R371) forms an electrostatic interaction with D311 of actin in the rigor conformation [7]. This model also showed that this myosin residue forms hydrogen bonds with the residues K328 and Q314 of actin. Additionally, a cryo-EM structure of actin-tropomyosin-myosin [30], incorporating the crystal structure of *Dictyostelium* myosin-IE [6], indicated the direct binding of this residue to the SD3 subunit of actin in the rigor conformation [30]. Cryo-EM structures of porcine cardiac actomyosin [5] and bovine β-myosin [3] displayed an electrostatic interaction between myosin R369 and tropomyosin, as well as electrostatic interactions between loop 4 and actin. The cardiac actomyosin structure also revealed an electrostatic interaction between nearby residue E371 of loop 4 and F-actin to reinforce the binding of the myosin 50 kDa upper subdomain to actin [5]. Finally, the model of Doran et al. [3] suggests that R369 additionally serves a critical role in displacing tropomyosin from the myosin-binding site on actin (Figure 1), which would also be important for stabilizing actomyosin interaction.

Since the R369H mutation in loop 4 reduces maximal actin binding of *Drosophila* myosin, we hypothesized that its expression in *Drosophila* would yield defects in organism locomotion. Indeed, *R369H/+* flies displayed dominant reductions in flight ability at 3 weeks of age (Figure 3B). It will be interesting to determine if clinical aberrations arise from the *R369H/+* genotype in human skeletal muscle myosins, as its presence is currently of an unknown significance [31]. Additionally, *Drosophila* genetic manipulation permitted the analysis of *R369H/R369H* homozygotes, where progressive defects in flight ability were observed from ages 2 days to 3 weeks (Figure 3A). Ultrastructural properties of IFMs are normal in 3-week-old *R369H/R369H* flies (Figure 4), suggesting that the severe reductions in flight ability at this age are not caused by structural deterioration of myofibrils. Jump distances were also reduced in *R369H/R369H* flies (Figure 3C). Overall, these data suggest that the R369 actin binding residue is critical for contractile function. In support of our hypothesis, a human DCM patient harboring the *R369Q*/+ *MYH7* genotype for β-cardiac myosin exhibited a deficit in fractional shortening (FS) [4].

We also assessed whether *R369H* flies exhibit cardiac defects, since the *R369Q/+* genotype is associated with dilated cardiomyopathy in humans [4]. Cardiac physiological parameters (Figure 5) and ultrastructural properties (Figure 6) were mostly unaffected by the R369H mutation, which may not be surprising since the mutation does not introduce a charge change as in the human condition. Interestingly, in *Drosophila*, the effects of the mutation are more severe in skeletal muscles compared to cardiac muscle, which may arise from differences in the amino acid backbone of the myosin isoforms that result from alternative splicing of transcripts from the single *Mhc* gene [32], and/or from differences in muscle physiological properties. In this regard, a number of mutations that severely affect the IFM induce relatively minor changes in the heart [15,33,34,35,36], as the IFM is a specialized, highly-ordered muscle that is exquisitely sensitive to mutation, and thus serves as an excellent system to identify even minor sarcomeric protein disruptions in vivo.

In summary, we exploited the *Drosophila* model system to elucidate the importance of a conserved myosin residue at the actin-binding interface. We found that a conservative R369H mutation impairs maximal actin binding and reduces skeletal muscle function (our results are summarized in Table 1). A better understanding of the intermolecular interactions between myosin and actin may yield insight into the mechanistic basis for myopathies caused by impaired actomyosin binding.

## 4. Methods

### 4.1. Generation of Mutant (Non His-Tagged) Fly Lines

A pCaSpeR vector containing a 19.2 kb *Mhc* 5′ fragment was digested with PstI and EagI [11]. A 3.1 kb fragment from this digest was gel isolated and ligated to a PstI-EagI site in a Bluescript KS vector. The resulting subclone was digested with NsiI and BglII. A 1.3 kb fragment was gel isolated and ligated to a NsiI and BglII site in a Litmus 28i vector. Oligonucleotide-directed mutagenesis (QuikChange kit, Agilent, Santa Clara, CA, USA) was performed to change the exon 8 codon corresponding to the *Mhc R369H* mutation using human β-cardiac myosin numbering (underlined) with the following forward (+) and reverse (−) primers:

(+): 5′-CAAGCAACGTGGTCACGAGGAGCAGGCTG-3′

(−): 5′-CAGCCTGCTCCTCGTGACCACGTTGCTTG-3′

The mutant subclone was sequenced for verification by Eton Bioscience (San Diego, CA, USA). Mutant subclones were inserted into larger intermediate subclones to generate a 5′ *Mhc* R369H subclone. Plasmids carrying the *Mhc* R369H 5′ fragment and wild-type *Mhc* 3′ were digested with EagI. The wild-type 3′ *Mhc* fragment was gel isolated and ligated into the EagI-digested R369H 5′ *Mhc* subclone to produce a final clone containing full-length R369H *Mhc* in a pCaSpeR *P* element vector. The final clone was purified using the Qiagen *QIAfilter Plasmid Maxi Kit and* sequenced by Eton Bioscience (San Diego, CA, USA) for verification.

A total of 20 transgenic mutant lines were generated by *P* element transformation by BestGene, Inc. (Chino Hills, CA, USA) [37]. Lines mapping to the third chromosome were crossed into the *Mhc*^10^ genetic background that is myosin heavy chain-null in IFMs and jump muscles [9]. Myosin heavy chain protein expression levels were determined by polyacrylamide gel electrophoresis by comparing the ratios of myosin to actin between mutant and *yw* (wild-type) controls [11]. Lines expressing wild-type levels of myosin heavy chain were selected for further experiments.

RT-PCR confirmed that *Mhc* transcripts from transgenic lines express the mutant transgene and were correctly spliced. For this purpose, RNA was isolated from the IFMs of 2-day-old flies using the RNeasy kit by Qiagen, Inc. (Hilden, Germany). cDNAs were generated using the ProtoScript cDNA synthesis kit by New England Biolabs (Ipswich, MA, USA) and amplified by PCR using the following primer pairs as previously reported [11]:

Exons 2–8:

(+) 5′-TGGATCCCCGACGAGAAGGA-3′

(−) 5′-GTTCGTCACCCAGGGCCGTA-3′

Exons 8–12:

(+) 5′-TCTGGATACCCAGCAGAAGCGT-3′

(−) 5′-GAGCTTCTTGAAGCCCTTACGG-3′

Exon 15:

(+) 5′-CTCAAGCTCACCCAGGAGGCT-3′

(−) 5′-GGGTGACAGACGCTGCTTGGT-3′

For simplicity, lines *R369H-1*, *R369H-16*, and *R369H-6* are referred to as *R369H-L1*, *R369H-L2*. and *R369H-L3.*

### 4.2. Steady-State ATPase Activity

Basal and actin-activated ATPase activity of myosin was determined with [γ-^32^P]-ATP [38]. Each assay sample was run in duplicate. Ca-ATPase activity was measured for 2 μg of full-length myosin in Ca-ATPase buffer (10 mM imidazole, pH 6.0, 100 mM KCl, 10 mM CaCl_2_) and 1 mM [γ-^32^P]-ATP at room temperature for 15 min. Mg-ATPase activity was measured for 2 μg of full-length myosin in Mg-ATPase buffer (10 mM imidazole, pH 6.0, 20 mM KCl, 0.1 mM CaCl_2_, 1 mM MgCl_2_) and 1 mM [γ-^32^P]-ATP at room temperature for 25 min in the presence of varying concentrations of chicken F-actin (0–2 μM). The reaction was stopped by addition of HClO_4_. After centrifugation, aliquots of the supernatant were extracted with a mixture of ammonium molybdate, HClO_4_ and isobutanol:benzene (1:1), as previously described [38]. Following phase separation, an aliquot of the organic phase was assessed by Cerenkov counting to determine ^32^Pi released during the reaction. After subtraction of basal ATPase activity (0 mM actin) for each sample, actin-activated ATPase activities were plotted vs. actin concentration, and the Michaelis–Menten equation was used to determine *V_max_* for actin-stimulated ATPase activity and *K_m_* for actin affinity relative to ATPase activity (N ≥ 3).

### 4.3. Generation of a Mutant His-Tagged Myosin Line and Protein Purification

We generated a His-tagged line for bulk myosin S1 isolation from IFMs in quantities sufficient for co-sedimentation assays, as reported previously [11]. A plasmid encoding an N-terminal His-tag, the *Actin88F* promoter, a tobacco etch virus protease recognition site, and full length myosin was digested with XbaI and SbfI [10]. A 6.3 kb fragment was gel isolated and ligated into a XbaI-SbfI site in the pLitmus vector to generate a pXbaSbf subclone. The pXbaSbf clone was digested with EcoRV and NcoI, and a 2.6 kb fragment was gel isolated and ligated into a EcoRV and NcoI site in the pLitmus vector to generate a pEcoRVNcoI subclone. Site-directed mutagenesis (QuikChange kit, Agilent, Santa Clara, CA, USA) was performed to change the exon 8 codon corresponding to the *Mhc R369H* mutation using human β-cardiac myosin numbering (underlined) with the following forward (+) and reverse (−) primers.

(+): 5′-CAAGCAACGTGGTCACGAGGAGCAGGCTG-3′

(−): 5′-CAGCCTGCTCCTCGTGACCACGTTGCTTG-3′

The mutated exon was verified by sequencing (Eton Bioscience, San Diego, CA, USA) and inserted into a larger pXbaSbf R369H cloning intermediate. The entire construct insert was removed and ligated into a pAttB vector containing the *miniwhite* (*w+*) eye color marker and the PhiC_31_ AttB recognition sites [10]. The final clone was purified and utilized for targeted insertion into the third chromosome by BestGene Inc. (Chino Hills, CA, USA) using the PhiC_31_ system [37]. One transgenic line was produced and crossed into a *Mhc*^10^ background that is myosin-null in IFMs [9]. Myosin protein is expressed in upper thoraces at levels ~80% relative to *yw* (wild-type) flies, as determined by polyacrylamide gel electrophoresis and densitometry [39].

RT-PCR of RNA isolated from upper thoraces was performed, as reported above, for non His-tagged fly lines using the following forward (+) and reverse (−) primers.

(+) 5′-TGGATCCCCGACGAGAAGGA-3′

(−) 5′-GTTCGTCACCCAGGGCCGTA-3′

The (−) end directed primer was used to generate cDNAs. RT-PCR products were sequenced by Eton Bioscience (San Diego, CA, USA) for verification. Bulk isolation of His-tagged mutant IFI and subsequent purification of the mutant motor domain was performed as previously reported [11].

### 4.4. Co-Sedimentation Assays

The binding affinity of S1 for F-actin was measured using the co-sedimentation approach, as previously described [11]. Briefly, S1 (1.5 μM) purified from His-tagged lines was added to phalloidin-stabilized F-actin (0, 0.4, 0.6. 0.8, 1, 1.2, 1.4, 1.6, 1.8, 2, 3, and 4 μM) in 25 μL co-sedimentation buffer (100 mM NaCl, 200 mM sodium phosphate buffer pH 7, 5 mM MgCl_2_, 4 mM DTT). Samples were incubated for 15 min at 4 °C and centrifuged (Beckman TLA-100.3 rotor, 70,000 RPM, 60 min, 4 °C). The insoluble portion containing actomyosin was resuspended in 25 μL co-sedimentation buffer and analyzed using SDS-polyacrylamide gel electrophoresis and densitometry. The bound fraction for each actin concentration was calculated as the density of S1 in the insoluble fraction relative to total protein content minus the fraction of pelleted material in S1-only controls. The bound fraction values over increasing F-actin concentrations were plotted and fit to a hyperbolic function to calculate the actin binding affinity (*K_d_*) and *B_max_* (N = 3).

### 4.5. Transmission Electron Microscopy of IFMs and Hearts

The thorax containing IFMs and the dorsal vessel containing the heart were dissected from female mutant and control lines, relaxed in a solution with 10 mM EGTA and fixed for transmission electron microscopy as previously reported [11]. Samples were fixed for ~16 h in primary fixative (3% formaldehyde, 3% glutaraldehyde in 0.1 M sodium cacodylate, pH 7.4) at 4 °C, washed 6 X in wash buffer (0.1 M sodium cacodylate, pH 7.4), fixed for 2 h in secondary fixative (1% OsO_4_, 0.1 M sodium cacodylate buffer pH 7.4, and 10 mM MgCl_2_) at 4 °C, and then washed 3 X in HPLC-H_2_O at 22–23 °C. Samples were dehydrated in increasing concentrations of acetone (25%, 50%, 75%, 95%, and 3X 100% anhydrous) for 30 min each and then infiltrated with increasing ratios of epoxy:acetone (1:3, 1:1, 3:1) for 2 h each. Samples were incubated in 100% epoxy for 16 h at 22–23 °C and then polymerized at 60 °C for 24 h under vacuum. Thin sections were generated using an ultramicrotome, stained for 20 min with 2% uranyl acetate, and imaged using a FEI Tecnai 12 transmission electron microscope. Sarcomere lengths were measured from longitudinally-oriented micrographs of IFMs using NIH ImageJ (N = 3, total of 50 sarcomeres per biological replicate, N), as previously reported [11]. The average distances between thick filaments were measured from transversely-oriented micrographs of IFMs using a custom-written Python script [11]. The distances between neighboring thick filaments were measured for a total of ≥700 thick filament centers per biological replicate (N = 3). To measure cardiac thickness, rectangular areas 10 microns in length that spanned the entire lengths, from the inner to outer cell membranes of the heart, were highlighted using Adobe Photoshop, as previously reported [11,18]. Highlighted images were imported into ImageJ to determine the area of that region. The area of each 10-micron length around the heart was divided by 10 to determine the average cardiac thickness (N = 3).

### 4.6. Flight and Jump Tests

Mutant and control females were maintained at 25 °C, acclimated to 22–23 °C for 1 h, and tested for flight ability at 22–23 °C as previously reported [11,12,33,40]. Each fly was released individually into a Plexiglas box illuminated at the top, and scored for its flight trajectory toward a light source, where upward flight (U) = 6, horizontal flight (H) = 4, downward flight (D) = 2, or no flight (N) = 0 [41]. Flight indices (FI) were calculated as (FI) = 6*U/T + 4*H/T + 2*D/T + 0*N/T, where ‘T’ represents the total number of flies for each trajectory listed above. A total of ≥100 flies were tested per line at each age. For jump tests, the wings were clipped, and each fly was placed on an inverted fly food vial with concentric rings drawn 0.5 cm apart to measure jump distance. Each fly was tactilely stimulated to jump, and the jump distance was recorded for 10 trials per fly. The average value for the top 3 of 10 jump distances were reported, and a total of N = 40 flies were tested per line/age.

### 4.7. Cardiac Physiology

Females were dissected in an oxygenated artificial hemolymph solution [108 mM NaCl, 5 mM KCl, 2 mM CaCl_2_, 8 mM MgCl_2_, 1 mM NaH_2_PO_4_, 4 mM NaHCO_3_, 5 mM HEPES (pH 7.1), 10 mM sucrose, and 5 mM trehalose] to expose the beating heart [42]. High-speed movies of heart contractions were recorded using a *Hamamatsu* Orca Flash 2.8 *CMOS or AOS Promon U750* camera for 30 s on a light microscope with a 10× objective. The Semi-automated Optical Heart Analysis (SOHA) software was employed to calculate heart diameters during systole and diastole, fractional shortening, heart rate, as well as systolic and diastolic intervals. A total of N = 40 flies were tested per line/age.

## Figures and Tables

**Table 1:** Summary of the effects of the R369H myosin mutation in *Drosophila*.

Characteristic	Effect
Myosin ATPase activity	Unaffected
Actin binding	Reduced
Indirect flight muscle function	Reduced
Jump muscle function	Reduced
Cardiac function	Unaffected
Cardiac and skeletal muscle structure	Unaffected

## Data Availability

Not applicable.

## Supplementary Materials

The following supporting information can be downloaded at: https://www.mdpi.com/article/10.3390/ijms23052533/s1. References [43,44] are cited in the supplementary materials.
