# Supplementary material for: The R369 Myosin Residue within Loop 4 Is Critical for Actin Binding and Muscle Function in Drosophila
Source: Int J Mol Sci. 2022 Feb 25;23(5):2533. doi: 10.3390/ijms23052533 (PMC8910226; doi:10.3390/ijms23052533)
Supplement: Supplementary file 1 [file ijms-23-02533-s001.zip › Trujillo et al R369H Table S1 Supplement.pdf]

Arg369 Missense Mutations in Striated Muscle Myosin Heavy Chain from the Genome Aggregation Database gnomAD v2.1.1

Human myosin heavy chain gene	Major muscle type expression [1]	Arg369 equivalent mutations	Missense mutant alleles/alleles screened [2, 3]	Allele frequency in descending order [2, 3]
MYH1	Late fetal skeletal	Arg372His	57/282794	2.02e-4
		Arg372Cys	42/282774	1.49e-4
MYH2	Fetal skeletal	Arg372Ser	1/31384	3.19e-5
		Arg372His	4/282840	1.41e-5
		Arg372Gly	2/251420	7.95e-6
		Arg372Pro	1/251440	3.98e-6
MYH3	Embryonic & fetal skeletal	None		
MYH4	Post-natal skeletal	None		
MYH6	α-cardiac (atria)	Arg370Trp	5/251328	1.99e-5
MYH7	β-cardiac (ventricles); slow skeletal	Arg369Trp	1/251380	3.98e-6
MYH7B	Specialized skeletal	None		
MYH8	Embryonic & fetal skeletal	Arg372His	7/251458	2.78e-5
		Arg372Cys	4/251450	1.59e-5
		Arg372Ser	2/251450	7.95e-6
MYH13	Extraocular skeletal	Arg371His	2/251354	7.96e-6
		Arg371Cys	1/251302	3.98e-6
MYH15	Specialized skeletal	None		
MYH16	Specialized skeletal	None		

1. Schiaffino, S.; Rossi, A. C.; Smerdu, V.; Leinwand, L. A.; Reggiani, C., Developmental myosins: expression patterns and functional significance. *Skeletal Muscle* **2015**, 5, 22.

2. Karczewski, K. J.; Francioli, L. C.; Tiao, G. *et al.* The mutational constraint spectrum quantified from variation in 141,456 humans. *Nature* **2020**, 581, 434–443.

3. The Genome Aggregation Database: https://gnomad.broadinstitute.org/
